# Robotic-assisted surgery and sentinel lymph node biopsy in morbidly obese endometrial cancer patients: a retrospective cohort study

**DOI:** 10.1007/s11701-026-03700-2

**Published:** 2026-07-21

**Authors:** Elina Kivekäs, Synnöve Staff, Minna M. Mäenpää

**Affiliations:** 1 Faculty of Medicine and Health Technology, Tampere University, Arvo Ylpön katu 34, 33520 Tampere, Finland; 2 Department of Obstetrics and Gynecology, Tampere University Hospital, Elämänaukio 1, 33520 Tampere, Finland; 3 FICAN Mid, PL 2000 Arvo Ylpön Katu 6, 33521 Tampere, Finland

**Keywords:** Endometrial neoplasms, Obesity, Robotic surgery, Sentinel lymph node, Survival, Surgical complications

## Abstract

Obesity is associated with a worse prognosis in endometrial cancer (EC) patients. While robotic surgery has overcome many technical challenges of morbid obesity, lymphadenectomies are still performed less frequently, leading to understaging and potential undertreatment in highly obese patients. We aimed to assess whether robotic-assisted laparoscopy (RAL) and the implementation of sentinel lymph node biopsy (SLNB) have enabled guideline-compliant nodal staging in morbidly obese patients. Surgical and oncological outcomes were also assessed. This retrospective single-center cohort study included all patients who underwent primary robotic surgery for EC at Tampere University Hospital, Finland, between March 2009 and December 2022. BMI stratified patients into morbidly obese (BMI ≥ 40 kg/m^2^) and non-morbidly obese (< 40 kg/m^2^) groups. Primary outcomes were nodal staging rates, bilateral sentinel lymph node (SLN) detection rates, and surgical complications. Cox regression analysis was used to assess survival outcomes. Of the 749 patients, 127 (17%) were morbidly obese and 622 (83%) had a BMI < 40. Following the implementation of SLNB, successful nodal staging was achieved in 87% of morbidly obese patients, with a bilateral SLN detection rate of 78%. Lymphadenectomies were performed less frequently in morbidly obese patients. Intraoperative and postoperative complication rates did not differ between BMI groups. No conversions were recorded in the final five years of the study. Higher BMI was associated with poorer overall survival (HR 1.04, 95% CI 1.02–1.07, p = 0.002), but not with progression-free survival or disease-specific survival. With robotic-assisted laparoscopy and SLNB, appropriate staging was achieved in most morbidly obese patients in this cohort.

## Introduction

Obesity is an independent risk factor for several types of malignancies, but in women, the strongest association is with endometrial cancer (EC). For every excess 5 kg/m^2^ rise in BMI, the risk of EC increases by approximately 50% [1, 2]. At least half of EC in the United States and the UK is thought to be attributable to obesity, and the lifetime risk for EC of a woman with a BMI > 40 kg/m^2^ is 10–15% [1, 3]. The incidence of EC has risen for decades alongside the increasing prevalence of obesity, with 420,000 new diagnoses made in 2022 [4, 5].

The surgical treatment of EC consists of hysterectomy and bilateral salpingo-oophorectomy, with nodal staging recommendations evolving substantially over the past 15 years. Early guidelines recommended selective nodal evaluation based on histology and depth of myometrial invasion, with pelvic lymphadenectomy as the primary staging procedure [6]. In the 2010 s, para-aortic lymphadenectomy was incorporated into clinical guidelines [7]. Later, sentinel lymph node biopsy (SLNB) was introduced as a possible alternative to lymphadenectomy, and the most recent clinical guidelines recommend offering SLNB as a method for nodal staging for all EC patients [8].

Obesity poses technical challenges to minimally invasive surgery, particularly in the performance of lymphadenectomies. While robotic surgery has overcome many technical challenges of morbid obesity, lymphadenectomies are still performed less frequently, leading to understaging and potential undertreatment in highly obese patients [9, 10]. Such limitations are thought to contribute, at least in part, to the poorer prognosis observed in obese individuals with endometrial cancer [11].

We aimed to evaluate the surgical and oncological outcomes of morbidly obese patients operated on a robotic platform. We also assessed the use of SLNB in the surgical management of morbidly obese patients.

## Materials and methods

### Study design

This single-center retrospective cohort study was conducted at the Department of Gynecology and Obstetrics, Tampere University Hospital, Tampere, Finland. The study was approved by the Institutional Review Board of Pirkanmaan hyvinvointialue (Pirkanmaa Wellbeing Services County) (approval ID: R23582R, decision § 163/2024). In accordance with Finnish legislation (the Act on the Secondary Use of Health and Social Data, 552/2019), ethical committee evaluation and written informed consent were not required. It was verified that no patients in the cohort had issued a data disclosure restriction.

**Fig. 1:**
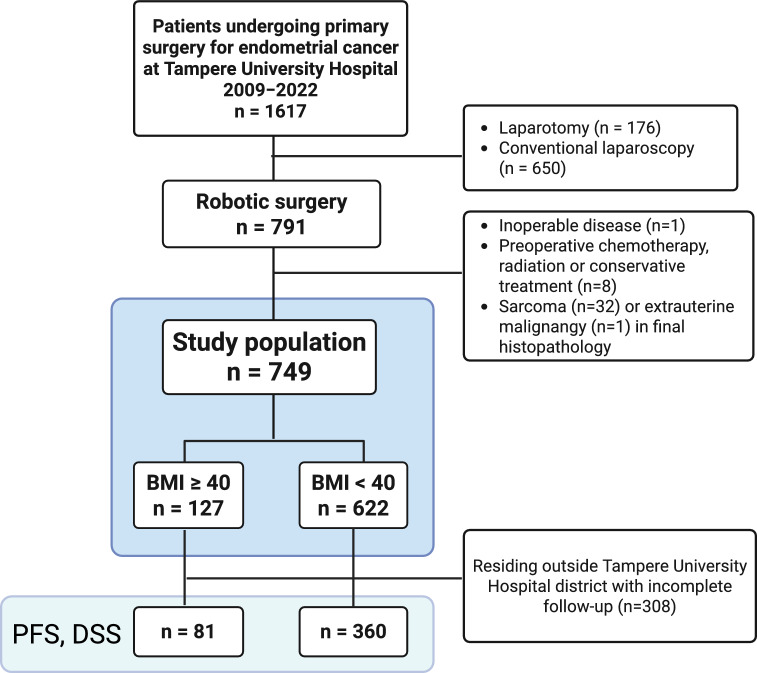
Flow chart of patient selection. Created with BioRender.com

Patients who underwent primary robotic-assisted laparoscopy (RAL) for EC at our institution between March 24th, 2009, and the end of December 2022 were included. Patients were excluded if they had received neoadjuvant chemotherapy or radiation, were found inoperable, or had extrauterine malignancy or carcinosarcoma on final histopathology. Patients were stratified by body mass index (BMI) into morbidly obese (BMI ≥ 40 kg/m^2^) and non-morbidly obese (< 40 kg/m^2^) groups. This BMI threshold is widely recognized as the threshold for morbid obesity and has been associated with substantially increased surgical challenges and conversion rates. As this was a retrospective registry-based study, no formal sample size calculation was performed; all eligible patients were included. The flowchart for patient selection is presented in Fig. 1.

Primary outcomes included surgical details and complications. Surgical details of interest were rates of lymphadenectomy (pelvic and para-aortic) and SLNB, bilateral SLN detection rate, conversions, and the success of the intended surgical extent. Surgical complications included intraoperative and 30-day postoperative (Clavien-Dindo) complications. Secondary outcomes were progression-free survival (PFS), disease-specific survival (DSS), and overall survival (OS).

At our institution, RAL has been part of standard practice since 2009. The proportion of RAL increased gradually over the study period. In the final years, half of the minimally invasive surgery (MIS) operations for EC were performed on the robotic platform. RAL was primarily selected for obese patients and those requiring comprehensive nodal staging. In the early years of the study, high-risk patients were managed with laparotomy, whereas low- and intermediate-risk patients underwent MIS. Morbidly obese patients eligible for MIS were operated on the robotic platform. Over the study period, the surgical approach for high-risk patients gradually shifted from laparotomy to RAL; consequently, in the later years, most high-risk patients were managed using RAL. Laparotomy was performed only for patients with advanced disease.

Regarding nodal staging, pelvic lymphadenectomy was the primary staging procedure until para-aortic lymphadenectomy was systematically adopted in 2013. The introduction of the da Vinci Xi platform in October 2017 enabled the implementation of SLNB; thereafter, all patients operated via RAL underwent SLNB. Initially, SLNB was performed concurrently with full lymphadenectomy in intermediate- and high-risk patients. Toward the end of the study period, SLNB alone was used for all patients except those with high-risk disease, who continued to undergo full lymphadenectomy. The pre-SLNB era was defined as surgery before the end of 2017, and the SLNB era as surgery from the beginning of 2018 onward, excluding the adoption phase of the procedure. SLNB was performed by injecting indocyanine green (ICG) into the uterine cervix at the 3 o’clock and 9 o’clock positions, administering 2 mL superficially and 2 mL deeply at each site. In cases where the sentinel lymph node was not detected, an ipsilateral pelvic lymphadenectomy was performed.

Preoperative high-risk features were defined as non-endometrioid or grade 3 endometrioid histology in preoperative sampling, or suspicion of deep myometrial invasion or lymphatic spread in preoperative imaging. Myometrial invasion was not assessed or was non-evaluable preoperatively in 21% of morbidly obese and 26% of non-morbidly obese patients, and computed tomography scan (CT) was not performed in 15% and 16% of patients, respectively. This reflects the selective use of imaging in the early years of the study period, as preoperative assessment of myometrial invasion and lymphatic spread with ultrasound, magnetic resonance imaging (MRI), and CT was routinely adopted from December 2011 onward.

Staging was defined as performed if the patient had undergone pelvic lymphadenectomy with or without para-aortic lymphadenectomy, SLNB with or without lymphadenectomy, or removal of a suspicious node. In the SLNB era, successful nodal staging was defined as either successful SLN mapping or, in the event of mapping failure, completion of lymphadenectomy. The FIGO 2009 staging guidelines were applied throughout the study period [6].

Survival analyses of progression-free survival and disease-specific survival were restricted to patients residing within the Tampere University Hospital district (n = 441) to ensure completeness of follow-up data. Given the centralized structure of cancer care in Finland, where patients with suspected recurrence are systematically referred to university hospitals, this approach was considered appropriate for fully capturing disease events. Progression-free survival was defined as the time from surgery to the first endometrial cancer recurrence or the end of follow-up. Disease-specific survival was defined as the time from surgery to death from endometrial cancer or the end of follow-up; patients who died of non-disease-related or unknown causes were censored at the time of death. Overall survival was defined as the time from surgery to death from any cause or the end of follow-up, whichever occurred first. The date of the last medical record review was used as the censoring date for patients without documented recurrence or death.

Complete data on patient demographics, preoperative evaluation, surgical details, histopathology, planned adjuvant therapy, and vital status were available for all patients. Data on postoperative complications, disease recurrence, and cause of death were limited among patients residing outside the Tampere University Hospital district. The number of available observations is indicated in the tables. The above-mentioned parameters were extracted from the medical database by the study investigators. The data were stored and analyzed in a secure platform provided by Tampere University Hospital. The STROBE guidelines for cohort studies were followed in reporting the results.

### Statistical analysis

Non-normally distributed variables were compared using the Mann–Whitney test. The Chi-square test or Fisher´s exact test was used for categorical variables. Multivariable Cox models identified risk factors for PFS, OS, and DSS. Variables associated with PFS, OS, or DSS at p < 0.05 in univariate analysis were included in the multivariable Cox models. BMI was retained in all models regardless of its univariate significance, as it was the primary variable of interest in this study and because morbidly obese and non-morbidly obese patients differed substantially in disease risk classification and age distribution. Cox regression for PFS and DSS was performed among patients residing within the Tampere University Hospital district (n = 441). All patients were included in the OS analysis. All tests were two-sided with significance set at p < 0.05, using SPSS (version 29.0; IBM, Armonk, NY).

## Results

The study included 749 patients who underwent primary RAL surgery for endometrial cancer between March 24th, 2009, and the end of December 2022. The body mass index (BMI) of the patients ranged from 17 to 77 kg/m^2^, while 127 (17%) were morbidly obese (BMI ≥ 40), and 622 (83%) had a BMI < 40. Morbidly obese patients were younger and had more comorbidities categorized by the Charlson Comorbidity Index. They also had a higher incidence of sleep apnea and previous deep vein thrombosis and were more frequently on anticoagulation therapy at the time of EC diagnosis. Final histology and stage also differed between the two BMI groups, with a higher prevalence of low-risk EC (51% vs 28%, p < 0.001) in the morbidly obese group, which was classified as endometrioid, grade 1–2, stage IA, with no lymphovascular space invasion. Among intermediate- to high-risk EC patients, the stage or administered adjuvant therapy did not differ between the groups. The description of patient demographics, final histopathology, and adjuvant therapy is provided in Table 1. The median follow-up time was 5.3 years (IQR 3.5–8.3) among morbidly obese patients and 5.5 years (3.1–8.8) among patients with BMI < 40 (p = 0.72).

**Table 1:** The demographic and disease characteristics of the patients

Characteristics	BMI < 40 n = 622		BMI ≥ 40 n = 127		p-value
	n	(%)	n	(%)	
Age, y, median, range	71	26–92	64	36–84	< 0.001
BMI, median, range	29	17–39	46	40–77	
Charlson comorbidity index					< 0.001
0	430	(69)	71	(56)	
1–2	189	(30)	51	(40)	
≥ 3	3	(1)	5	(4)	
Sleep apnea	25	(4)	26	(21)	< 0.001
Previous deep vein thrombosis	32	(5)	16	(13)	0.002
Previous malignancy	78	(13)	12	(9)	0.33
Anticoagulation therapy	66	(11)	23	(18)	0.02
Final histopathology					
Endometrioid	473	(76)	118	(93)	< 0.001
High-grade	226	(36)	25	(20)	< 0.001
Lymphovascular space invasion	275	(44)	37	(29)	0.002
Myometrial invasion ≥ 50%	276	(44)	38	(30)	0.003
Low-risk EC*	171	(28)	65	(51)	< 0.001

Intermediate- to high-risk EC	n = 451		n = 62		
Stage					0.27
IA	109	(24)	14	(23)	
IB	136	(30)	25	(40)	
II	49	(11)	8	(13)	
III–IV	157	(35)	15	(24)	
Adjuvant therapy	427	(95)	59	(95)	1.0
Type of adjuvant therapy					0.26
Brachytherapy	147	(34)	22	(37)	
External radiation	34	(8)	8	(14)	
Chemotherapy ± radiation	246	(58)	29	(49)	

*Stage IA, endometrioid, G1–2, no lymphovascular space invasion

The surgical details of the whole study population are presented in Table 2. During the study period, nodal staging was performed in 68% of morbidly obese and 95% of non-morbidly obese patients (p < 0.001). Because nodal staging recommendations evolved over the study period, and morbidly obese patients had a markedly higher prevalence of low-risk disease, withholding nodal staging was guideline-compliant for many morbidly obese patients in the early study period. To isolate genuine understaging from guideline-appropriate non-staging, nodal staging rates were therefore evaluated specifically among patients with a confirmed high-risk feature on preoperative work-up — a group for whom staging would have been indicated even in the early study period. In the preoperative assessment, high-risk features were observed less commonly in morbidly obese patients (45% vs. 74%, p < 0.001). Among these higher-risk patients, nodal staging was performed in 81% versus 96% of the patients (p < 0.001). In the morbidly obese group, both pelvic and para-aortic lymphadenectomies were performed significantly less (p < 0.001), and fewer nodes were retrieved in para-aortic lymphadenectomy (p = 0.003). The intended surgical extent was not achieved in 23 (18%) of morbidly obese patients and 29 (5%) of non-morbidly obese patients (p < 0.001). Conversions to laparotomy occurred more often in the morbidly obese group (4%, vs. 1%, p = 0.02). Eight of the eleven conversions occurred between 2009 and 2013, while no conversions were recorded after 2017. Median hospital stay was 1 day in both groups. Intraoperative and 30-day postoperative complication rates did not differ between BMI groups. Among patients with available complication data, the overall Clavien–Dindo complication rate was lower in patients staged with SLNB alone (n = 101) than in those who underwent lymphadenectomy (n = 443) (20% vs. 44%, p < 0.001). Among morbidly obese patients, the corresponding complication rates were 26% (9/35) and 47% (16/34) (p = 0.08).

**Table 2:** Surgical details of the patients during the whole study period

Characteristics	BMI < 40 n = 622		BMI ≥ 40 n = 127		p-value
	n	(%)	n	(%)	
Nodal staging performed	591	(95)	86	(68)	< 0.001
High-risk feature in preoperative analysis*	461	(74)	57	(45)	< 0.001
Nodal staging performed	443	(96)	46	(81)	< 0.001
Surgical procedure					
Pelvic lymphadenectomy	502	(81)	41	(32)	< 0.001
Lymph nodes removed, median, IQR	23	17–30	28	14–34	0.31
Para-aortic lymphadenectomy	385	(62)	25	(20)	< 0.001
Lymph nodes removed, median, IQR	13	9–18	8	5–14	0.003
Sentinel lymph node biopsy	235	(38)	60	(47)	0.05
Conversion to laparotomy	5	(1)	5	(4)	0.02
Intended surgical extent not achieved	29	(5)	23	(18)	< 0.001
Intraoperative complication	28	(5)	4	(3)	0.49
Blood loss, mL, median, IQR	50	30–100	50	50–150	0.09

Postoperative complications, 30-day (Clavien Dindo)**	n = 505		n = 108		0.43
Grade I–II	165	(33)	41	(38)	
Grade III–V	36	(7)	5	(5)	

*High-grade histology, or suspicion of deep myometrial infiltration (> 50%) or lymphatic spread

**Data available

After the implementation of SLNB, between 2018 and 2022, all morbidly obese patients and 93% of the non-morbidly obese patients underwent SLNB. The bilateral SLN detection rate did not differ between the BMI groups (78% vs. 86%, p = 0.16). An empty SLN specimen was observed in 3% of patients in both groups. Among patients with failed SLN mapping, ipsilateral lymphadenectomy was performed in 39% and 91% patients, respectively (p < 0.001) (Table 3). During the SLNB era, successful SLNB or lymphadenectomy was performed in 87% of morbidly obese and 98% of non-morbidly obese patients (p < 0.001). In the pre-SLNB era, 39% (26/67) of morbidly obese patients underwent nodal staging overall, rising to 59% (16/27) among those with high-risk features on preoperative evaluation.

**Table 3:** Nodal staging details in the era of sentinel lymph node biopsy

Staging procedure in the SLNB era 2018–2022	BMI < 40 n = 251		BMI ≥ 40 n = 60		
	n	(%)	n	(%)	
No staging	3	(1)	0	(0)	1.0
Pelvic lymphadenectomy	162	(65)	17	(28)	< 0.001
Para-aortic lymphadenectomy	156	(62)	11	(18)	< 0.001
Sentinel lymph node biopsy	233	(93)	60	(100)	0.03
Detected bilaterally	201	(86)	47	(78)	0.16
No lymph nodes in dissection	8	(3)	2	(3)	0.98

Sentinel node detection failure	n = 32		n = 13		
Ipsilateral lymphadenectomy performed	29	(91)	5	(39)	0.001
Successful SLNB or lymphadenectomy	245	(98)	52	(87)	< 0.001

PFS and DSS analyses were performed among patients residing in the Tampere University Hospital district (n = 441). In the multivariable Cox regression analysis, body mass index was not associated with poorer PFS (HR 1.01, 95% CI 0.98–1.04, p = 0.48) or DSS (HR 1.00, 95% CI 0.96–1.03, p = 0.83) after adjustment for tumor characteristics and stage. Among all 749 patients, higher BMI was associated with worse OS (HR 1.04, 95% CI 1.02–1.07, p = 0.002). High-grade histology was an independent risk factor for all survival outcomes: stage > II for DSS; age for OS; and lymphovascular space invasion for PFS (Table 4).

**Table 4:** The Cox regression analysis of oncological survival outcomes

Covariate	Univariate analysis			Multivariable analysis		
	HR	95% CI	p-value	HR	95% CI	p-value
*Progression-free survival, n = 441*						
BMI	0.98	0.96–1.01	0.21	1.01	0.98–1.04	0.48
Age	1.02	1.00–1.05	0.03	1.01	0.99–1.04	0.36
LVSI +	3.04	1.99–4.66	< 0.001	1.79	1.05–3.06	0.03
High grade	3.23	2.14–4.89	< 0.001	2.53	1.62–3.95	< 0.001
Myometrial invasion > 50%	2.31	1.52–3.51	< 0.001	1.45	0.89–2.37	0.13
Stage > II	2.87	1.88–4.38	< 0.001	1.44	0.87–2.34	0.16
*Disease-specific survival, n = 441*						
BMI	0.98	0.95–1.01	0.26	1.00	0.96–1.03	0.83
Age	1.04	1.01–1.06	0.01	0.98	0.95–1.01	0.22
LVSI +	4.05	2.40–6.83	< 0.001	2.12	0.95–4.75	0.07
High grade	5.89	3.51–9.84	< 0.001	3.47	1.97–6.11	< 0.001
Myometrial invasion > 50%	3.09	1.86–5.15	< 0.001	0.85	0.39–1.84	0.85
Stage > II	3.80	2.35–6.18	< 0.001	2.08	1.09–3.98	0.03
*Overall survival, n = 749*						
BMI	1.01	0.98–1.03	0.69	1.04	1.02–1.07	0.002
Age	1.05	1.03–1.08	< 0.001	1.05	1.03–1.08	< 0.001
LVSI +	2.39	1.65–3.47	< 0.001	1.36	0.84–2.18	0.21
High grade	2.93	2.03–4.22	< 0.001	2.50	1.67–3.71	< 0.001
Myometrial invasion > 50%	2.20	1.52–3.18	< 0.001	1.41	0.91–2.18	0.13
Stage > II	2.58	1.73–3.83	< 0.001	1.56	0.98–2.45	0.06

LVSI = lymphovascular space invasion

## Discussion

### Summary of main results

In this large single-center retrospective study, we report the surgical and oncological outcomes of patients with EC who underwent RAL, with a focus on those who were morbidly obese (BMI ≥ 40). The main results were as follows. Following the implementation of SLNB, successful nodal staging was achieved in 87% of morbidly obese patients, with a bilateral SLN detection rate comparable to that of non-morbidly obese patients. Throughout the study period, lymphadenectomy was performed less frequently in morbidly obese patients, including when SLN mapping failed, and fewer para-aortic lymph nodes were retrieved compared to non-morbidly obese patients. Obesity was not associated with increased intraoperative or postoperative complications, and conversions to laparotomy were rare. Higher BMI was associated with worse OS but not with worse PFS or DSS.

### Results in the context of published literature

RAL has emerged as the preferred surgical modality for morbidly obese EC patients, achieving higher rates of complete nodal staging compared to open surgery or conventional laparoscopy [12, 13]. Nevertheless, lymphadenectomy rates remain lower in morbidly obese patients even in RAL settings [9, 14, 15]. However, it is often unclear whether low staging rates reflect a lack of clinical indication, unsuccessful attempts, or procedures not performed despite indication. An exception was reported in a high-volume single-center study of 76 morbidly obese EC patients operated via RAL by a single surgeon, where successful pelvic and para-aortic lymphadenectomies were achieved in 96% and 75% of patients, respectively, with no conversions [13]; however, the generalizability of these results to multi-surgeon settings may be limited.

In our study, the higher prevalence of low-risk disease among morbidly obese patients explains some part of the lower nodal staging rates. However, in the pre-SLNB era, only 59% of patients with a high-risk feature in preoperative evaluation underwent nodal staging. Additionally, the intended surgical extent was not achieved in one-fifth of morbidly obese patients. Together, these findings suggest that inadequate staging in morbidly obese patients may be driven, at least in part, by genuine surgical challenges, although clinical decision-making related to comorbidity burden cannot be excluded.

Following SLNB implementation, nodal staging rates among morbidly obese patients increased markedly, as all morbidly obese patients underwent SLNB. However, nodal staging was successful only in 87% of morbidly obese patients, as lymphadenectomy was not always performed in cases of SLN detection failure. The bilateral SLN detection rate among morbidly obese patients was higher (78%) than those reported in previous studies, which have described rates of 54–64% in patients with a BMI ≥ 40 [16–18]. Of these, Vargiu et al. [18] also reported an empty-packet dissection rate of 7.5%, considerably higher than 3% observed in our cohort.

In a systematic review and meta-analysis by Cusimano et al. [16], the conversion rate of morbidly obese (BMI ≥ 40) patients with EC treated via RAL was also found to be 3.8%. In our study, eight out of the eleven conversions occurred during the first five years, with none happening in the last five years (2018–2022). This trend may reflect the surgeons’ learning curve, as half of the morbidly obese patients underwent surgery after 2017. The rates of intraoperative and 30-day postoperative surgical complications were similar between the BMI groups, as noted also in previous studies [16, 17].

Obesity has been linked to poorer prognosis in EC, with higher BMI associated with increased recurrence, overall mortality, and, in some studies, worse DSS [11, 18]. In our study, higher BMI was associated with worse OS but not with PFS or DSS, suggesting that excess mortality in morbidly obese EC patients is primarily driven by obesity-related comorbidities rather than EC itself — a finding consistent with population-based data where cardiovascular disease was shown to be the leading cause of death in EC patients [19].

Beyond staging considerations, the robotic platform may offer a further advantage in the surgical management of morbidly obese EC patients by enabling combined treatment of the oncological and metabolic components of their disease. A recent pilot study demonstrated the feasibility of performing robotic-assisted hysterectomy and bariatric surgery concurrently in a single surgical session, with acceptable intraoperative and 30-day morbidity [20]. A subsequent review similarly proposed combining bariatric and oncological surgery to improve long-term outcomes in obese EC patients, given the shared benefit of sustained weight loss for both cancer control and metabolic health [21]. Our cohort did not include such combined procedures, and this approach requires further prospective evaluation; nevertheless, it may represent a promising future avenue for addressing both the surgical challenges and the metabolic comorbidities associated with morbid obesity in this population.

### Strengths and weaknesses

The strengths of the study are the comprehensive population-based cohort and a reliable dataset. This study included all minimally invasively treated morbidly obese EC patients in our tertiary hospital district. Due to the structure of the Finnish healthcare system, all EC patients within the healthcare district are operated in a single center. Hence, the study population is unselected, for example, with respect to socioeconomic background. During the study period, robotic surgeries for EC were conducted by a select group of experienced gynecologic oncologists, with 4 surgeons performing 87% of the surgeries, thereby minimizing inter-surgeon variability and ensuring consistent surgical quality. The follow-up loss for patients residing within the Tampere University Hospital district was negligible.

The weaknesses of our study are the heterogeneity among the study groups, the retrospective design, and the relatively low number of morbidly obese patients. At the time of the study, half of the EC surgeries at our hospital were performed using RAL, and the other half were performed with conventional laparoscopy. The RAL cohort mainly included obese patients and those with high-risk disease. Therefore, the disease profile differed markedly between the groups, and the need for lymphadenectomies also varied significantly. Additionally, given the imbalance in high-risk EC characteristics between the study cohorts, the survival analyses should be interpreted cautiously. Another weakness is that the data on recurrence, causes of death, and postoperative complications were incomplete in patients who were followed up outside the Tampere University Hospital district. We did not include patients operated on using conventional laparoscopy or laparotomy. However, we do not consider it a weakness since all morbidly obese EC patients suitable for MIS were operated on using RAL during the study period.

Treatment protocols evolved over the study period, particularly regarding nodal staging. Therefore, it was not possible to assess whether nodal staging was performed in accordance with treatment guidelines at the time. Preoperative imaging was not performed uniformly across the study period, as routine MRI and CT evaluation were not introduced until December 2011, which may have affected preoperative risk stratification in the early cohort. Some patients categorized as not having a high-risk feature may, in fact, have had unrecognized high-risk disease.

## Conclusion

These findings suggest that RAL is feasible in morbidly obese patients and that, combined with SLNB, guideline-compliant nodal staging may be achievable in most patients of this surgically challenging population. As accumulating evidence indicates that SLNB alone may be a viable staging option even for high-grade EC [22], the need for lymphadenectomy may substantially decrease in the future. Nevertheless, for morbidly obese patients in whom sentinel lymph node mapping fails, performing a full lymphadenectomy continues to pose a surgical challenge.

## Data Availability

The data supporting the data of this study are not publicly available due to patient privacy regulations, but are available from the corresponding author upon reasonable request and with permission from the Institutional Review Board.
